# Transcatheter Repair of Mitral Valve Leaflet Perforation Using Occluder Plug

**DOI:** 10.1016/j.jaccas.2025.103421

**Published:** 2025-03-26

**Authors:** Qi Xuan Ang, Gabriel Panama, Mohammad Saad Salam, Zan Siddiqi, Shaurya Srivastava, Mohammed Qintar

**Affiliations:** a Department of Medicine, Michigan State University/University of Michigan Health Sparrow Hospital, Lansing, Michigan, USA; b Department of Cardiology, Michigan State University/University of Michigan Health Sparrow Hospital, Lansing, Michigan, USA

**Keywords:** Amplatzer Duct Occluder II, mitral valve, occluder plug, percutaneous, perforation, transcatheter

## Abstract

Mitral valve leaflet perforation (MVLP) is typically managed through surgical intervention and can be technically challenging among nonsurgical candidates. Thus, techniques involving off-label use of occluder devices in percutaneous mitral leaflet repair have been developed. The authors report a case series of MVLP repaired with an Amplatzer Duct Occluder II Plug. This case series suggests that transcatheter closure with an Amplatzer Duct Occluder II plug is a promising alternative to surgical repair for anterior mitral leaflet perforation, with favorable mid-term outcomes. We also performed a literature review of transcatheter repair of MVLP using different techniques.

## Graphical Abstract

**Figure:**
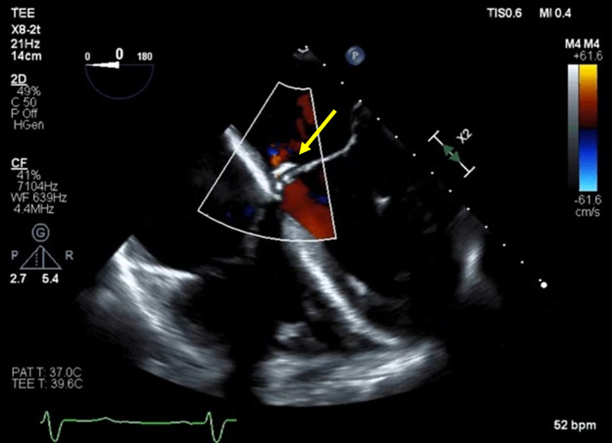

The advancement of structural interventional cardiology has progressed remarkably over the past decade, notably in its approach to addressing mitral valve pathology via mitral transcatheter valve edge-to-edge repair (TEER) for mitral regurgitation (MR).^1^ However, managing mitral leaflet perforation (MVLP) in nonsurgical candidates remains challenging owing to limited available literature and guidelines and a lack of devices engineered specifically for MVLP. To address these issues, transcatheter repair has been performed off-label using occluder devices. In this case series, we share our experience using an Amplatzer Ductal Occluder II (ADO II) (Abbott) to manage MVLP. Additionally, we performed a comprehensive literature review of transcatheter repair of MVLP using various techniques and devices.

**Learning Objectives**

• To explore the feasibility and effectiveness of transcatheter closure of MVLP with occluder devices.
• To investigate the short/mid-term outcomes of patients who underwent transcatheter closure of MVLP with ADO II plug.
• To perform a comprehensive literature review of the available techniques and devices used to repair MVLP.

## Case 1

A 79-year-old man with a past medical history of surgical bioprosthetic aortic valve replacement, coronary artery disease with multiple prior percutaneous coronary interventions, insulin-dependent type 2 diabetes mellitus, and end-stage renal disease on hemodialysis presented with acute right-sided weakness and was diagnosed with acute ischemic stroke, secondary to septic emboli from *Streptococcus mitis* endocarditis. Transesophageal echocardiography (TEE) demonstrated a perforation at the anterior leaflet of the mitral valve with severe MR (Videos 1 and 2). Cardiac computed tomography scan showed MVLP at the A3 segment, measuring 8 mm × 12 mm. His hospital course was complicated with acute hypoxic respiratory failure secondary to acute congestive heart failure. Considering his complicated surgical course after prior post-surgical aortic valve replacement and after consultation with our cardiothoracic surgery team he was deemed high risk for surgical procedures, and the patient chose to undergo transcatheter repair.

**Figure 1:**
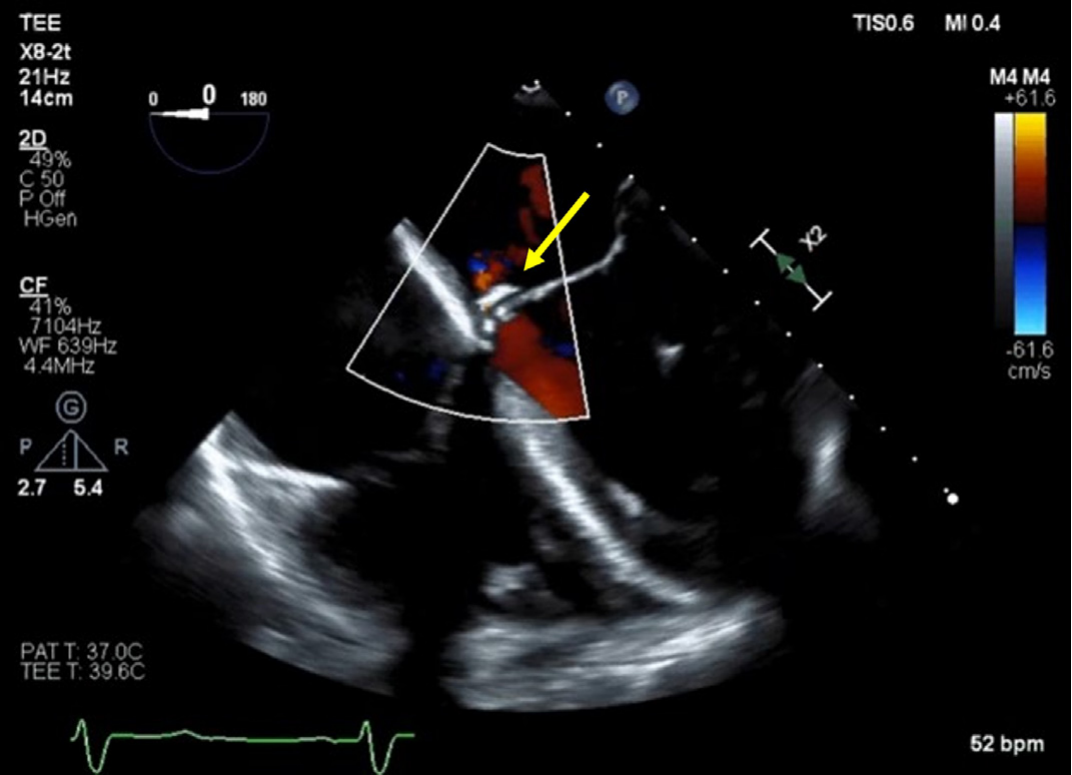
Case 1 Postprocedural transesophageal echocardiography (TEE) showed significant improvement of MR in the perforated area (yellow arrow, Amplatzer Duct Occluder II plug).

The patient continued on antimicrobial therapy for endocarditis for an additional 5 weeks following a series of negative blood cultures. He was scheduled for outpatient elective transcatheter repair with an ADO II Plug (Abbott) under general anesthesia and TEE guidance. After performing trans-septal puncture using standard technique, a steerable medium-curve Agilis catheter (Abbott) was advanced into the left atrium over a 1 cm, soft tip, super stiff Amplatzer wire. Using a mother–daughter technique inside the Agilis catheter (a Berenstein 4F 140-cm catheter [Merit Medical] inside a 6F MP guide); a 400-cm angled stiff Glidewire (Terumo) was advanced through the perforation into the left ventricle. A 6 mm × 4 mm ADO II plug was meticulously deployed across the perforation through the MP guide (Videos 3 and 4), Postdeployment TEE interrogation showed very trivial residual MR (Video 5, Figure 1). The patient tolerated the procedure without immediate complications and was discharged 1 day later. The patient had no major adverse events within 6 months following the procedure with NYHA functional class I to II.

### Equipment List

• TEE
• 6F sheath
• Baylis needle and sheath
• 14F DrySeal sheath (Gore Medical)
• Steerable Agilis sheath (Abbott)
• 4F Berenstein catheter (Merit Medical)
• 6F guide
• 400-cm angled stiff Glidewire (Terumo)
• 6 mm × 4 mm ADO II Plug (Abbott)

## Case 2

A 38-year-old woman with a past medical history of systemic lupus erythematosus and recent type A aortic dissection status post emergent Bentall procedure with an On-X mechanical aortic valve replacement presented with massive edema (anasarca) and severe NYHA functional class IV dyspnea. TEE demonstrates severe MR owing to 2 pathologies: 1) posterior mitral leaflet overriding the anterior leaflet; and 2) perforation at the base of the anterior mitral valve leaflet (Video 6). The patient was deemed high risk for redo surgical intervention owing to a recent history of complicated postsurgical course after her prior Bentall procedure.

After heart failure optimization, transcatheter repair of the mitral valve with M-TEER using MitraClip and repair of anterior MVLP using ADO II Plug was performed under general anesthesia and TEE guidance. After performing trans-septal puncture using standard technique, a steerable MitraClip delivery system (Abbott) was then advanced to the left atrium. A MitraClip device was deployed in the A2-P2 position with residual trivial MR (Video 7).

**Figure 2:**
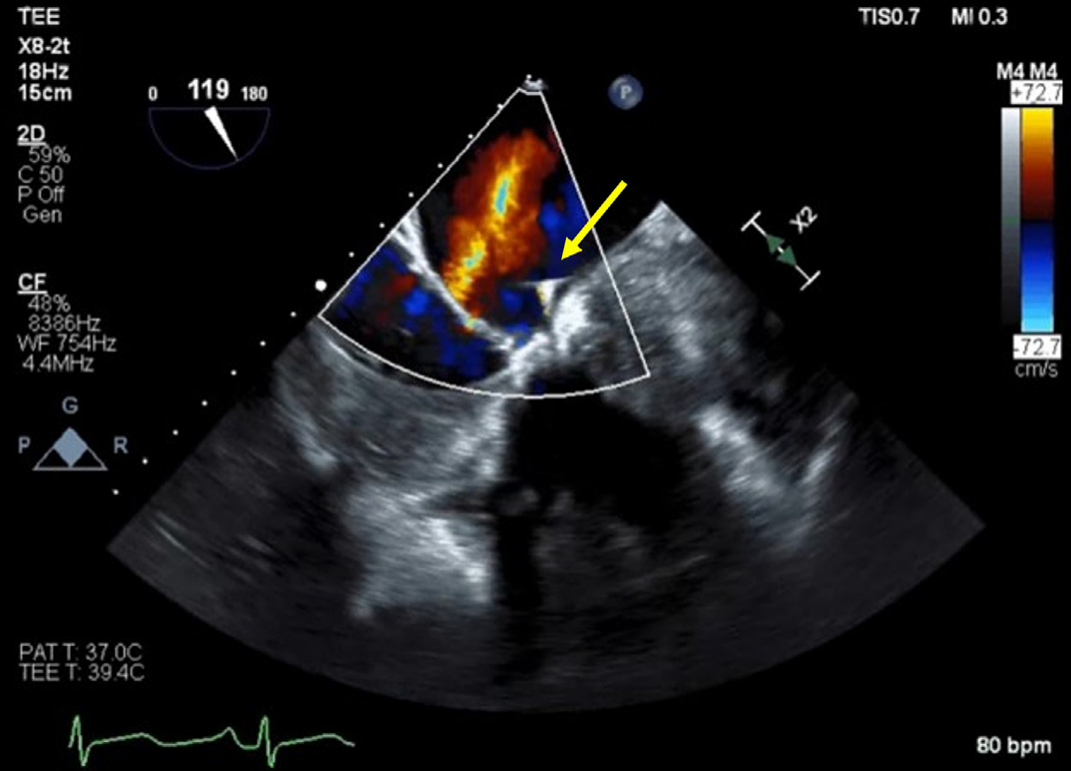
Case 2 Postprocedural transesophageal echocardiography (TEE) showed resolution of mitral regurgitation in the perforated area (yellow arrow, Amplatzer Duct Occluder II plug).

The MitraClip delivery system was withdrawn, and a 24F DrySeal sheath (Gore Medical) was placed via the right femoral vein. A steerable medium-curl Agilis sheath (Abbott) with a 4F Berenstein catheter (Merit Medical) was induced via femoral access. The valvular perforation was wired with a stiff angled Glidewire (Terumo), which was exchanged with a Confida wire (Medtronic). Over the Confida wire, a dedicated Torqvue delivery sheath was placed across the peroration, and then a 6 mm x 4 mm ADO II was deployed across the perforation (Video 8). Postprocedural TEE interrogation showed complete resolution of MR in the perforated area (Video 9, Figure 2).

She tolerated the procedure without immediate complications and was discharged 1 day later. For 6 months after the procedure, the patient had no major adverse events and had a functional status of NYHA functional class I and II.

## Literature Review

With the lack of guidelines and standardization of the procedure, literature review demonstrated a few different techniques and device usage, with each case presenting different approaches and challenges (Table 1). In Table 2, we summarize the common devices used. There have been cases of younger patients, aged 16 and 19 years,^2^^,^^3^ requiring MVLP plugging. Both cases shared similarities in terms of the history of surgical repair for congenital heart disease and the development of MVLP later in life. In both instances, the antegrade transseptal puncture approach was technically difficult, leading to the use of the retrograde transaortic approach.

**Table 1:** Literature Review of Different Publications

First Author	Demographics	Approach	Type of Device	Indication	Complications
Tafa et al^6^	(n = 16) Mean age: 55 y	n = 7 (Transaortic) n = 9 (Transapical)	n = 10 (ASO) n = 6 (AVP-II)	n = 9 (MVLP) n = 7 (postclip residual MR)	
Eng et al^4^	N = 4 Case 1: Age 88 y Case 2: Age 66 y Case 3: Age 66 y Case 4: Age 77 y	Case 1: Transseptal Case 2: Transapical Case 3: Transapical	Case 1: Amplatzer VSD and ADO II Case 2: 18 mm Amplatzer cribriform septal occluder Case 3: Cardioform septal occluder Case 4: Cardioform septal occluder	Case 1: post-clip residual MR Case 2: MVLP Case 3: MVLP Case 4: Postclip residual MR	Case 2: embolization of ADO II, GORE Cardioform septal occluder with deformation in the proximal disc Case 4: Device embolization
Kubos et al^5^	n = 9 Mean age: 79 y	Transseptal	ADO II	Postclip residual MR	Device embolization
Addis et al^7^	n = 1 Age 88 y	Transseptal	Amplatzer VSD	Co-deployment for MVLP and severe MR	
Carretero Bellon et al^2^	n = 1 Age 16 y	Transaortic	ADO II	MVLP	
Sengun et al^3^	n = 1 Age 19 y	Transaortic	ADO II	MVLP	
Lai et al^10^	n = 1 Age 45 y	Trans-septal	ADO II	Co-deployment for MVLP and severe MR	

ADO = Amplatzer Ductal Occluder; ASO = Amplatzer Septal Occluder; AVP-II = Amplatzer Vascular Plug II Occluder; Amplatzer VSD = Amplatzer Ventricular Septal Defect Occluder; MR = mitral regurgitation; MVLP = mitral valve leaflet perforation.

**Table 2:** Different Types of Occluder Devices Used for Mitral Valve Leaflet Perforation or Postclip Residual Regurgitation

Amplatzer Ductal Occluder II (Abbott)
Amplatzer Septal Occluder (Abbott)
Amplatzer Vascular plug II (Abbott)
Amplatzer Ventricular Septal defect (Abbott)
CARDIOFORM Septal Occluder (Gore Medical)

The most common reported complication was device embolization.^3^^,^^4^ Kubo et al^5^ reported a case of device embolization into the ostium of the right coronary artery presenting as an ST-segment elevation myocardial infarction after the procedure.

As mentioned previously, occluder devices have been used in residual regurgitation after TEER.^4^, ^5^, ^6^, ^7^ Addis et al^7^ and Eng et al^4^ experienced a unique situation in which post-TEER residual MR was noted, and an occluder device was deployed during the same procedure. In contrast to other reported cases, Lai et al^8^ demonstrated the use of an occluder device to plug a MV perforation with a telescoping delivery system followed by TEER.

## Discussion

The off-label use of occluder devices to treat paravalvular leaks has previously proven successful, with a large case series of 115 patients showing a survival rate of 64.3% from all-cause mortality.^9^ Its role has expanded into other applications, including: 1) MVLP; 2) residual regurgitation post-TEER in cases where regurgitation persists between 2 MitraClips; or 3) when regurgitation exists at the commissures.^6^

The application of the ADO II plug to repair MVLP is based on its design (tight braiding from 144 nitinol wires to withstand high-pressure systems) and proven efficacy in closing patent ductus arteriosus.^10^ The ADO II is a self-expanding device consisting of 2 nitinol wire mesh disks with an interconnecting waist, which ensures secure positioning within the perforation.^9^ It enables insertion at different angles by swiveling around the central part, and its symmetrical design permits its delivery from either an arterial or venous approach. Careful consideration needs to be made to ensure the success of ADO II plug closure, including: 1) variations in valvular perforation and mitral valve anatomy; 2) selection of size of ADO II plug, which comes in multiple sizes with waist diameters of 3, 4, 5, or 6 mm, and lengths of 4 mm or 6 mm, with the retention disk 6 mm larger than the waist; 3) guidance of imaging modalities; and 4) expertise in transcatheter repair.

The 6-month clinical outcomes in our case series were favorable, with both patients experiencing a significant decrease in MR to trace residual and symptomatic improvement. Moreover, the use of ADO II is associated with very low hemolysis rates owing to its tight braiding design to withstand high-pressure systems, making it the first choice if the size is favorable. The most common complication with ADO II is device embolization, likely owing to undersizing the ADO II. Thus, we recommend multimodality imaging sizing to ensure enough oversizing. There were no major complications, such as systemic embolization, hemolytic anemia, cardiac arrest, or leaflet failure. Although most evidence is based on case reports or case series, large-scale observational studies and randomized controlled trials are necessary to evaluate the long-term outcomes and safety of such an approach.

## Conclusions

The short-term and mid-term safety and efficacy of occluder devices for MVLP in nonsurgical candidates have been demonstrated in multiple case reports. Despite its promising short and mid-term outcomes, large-scale observational studies and randomized controlled trials are necessary to evaluate the long-term outcomes and safety of such an approach.

## Take-Home Message

The short-term and mid-term safety and efficacy of occluder devices for MVLP in nonsurgical candidates are promising; however, the technique's long-term durability, effectiveness, and safety still need to be studied further.

## Appendix

Video 1 Transesophageal echocardiography in mid-esophageal 4-chamber view showing MVLP in A3 position with severe eccentric jet mitral regurgication.

Video 2 Transesophageal echocardiography in 3-dimensional echocardiography and surgical view of the mitral valve showing a perforation at A3 with a severe regurgitant jet on color Doppler.

Video 3 Fluoroscopy image showing device delivery and release across the perforation.

Video 4 Transesophageal echocardiography in X-plane imaging showing the occluder device in the perforation.

Video 5 Transesophageal echocardiography showing the Amplatzer Duct Occluder II is in place at the perforation in A3 with no regurgitant jet appreciated and a normal final transmitral gradient.

Video 6 Transesophageal echocardiography showing severe mitral regurgitation (MR) from 2 pathologies: (1) A2-P2 severe MR and (2) a perforation at the base of the A2 leaflet.

Video 7 Transesophageal echocardiography in commissural view showing MitraClip in A2/P2 position. Reduction in mitral regurgitation to trace-mild.

Video 8 Fluoroscopy image showing device delivery and release across the perforation.

Video 9 Transesophageal echocardiography showing the Amplatzer Duct Occluder II plug in the perforation site with no residual mitral regurgitation jet appreciated at the perforation site.
